# Polymer/Nanocrystal Hybrid Solar Cells: Influence of Molecular Precursor Design on Film Nanomorphology, Charge Generation and Device Performance

**DOI:** 10.1002/adfm.201403108

**Published:** 2014-11-25

**Authors:** Andrew J MacLachlan, Thomas Rath, Ute B Cappel, Simon A Dowland, Heinz Amenitsch, Astrid-Caroline Knall, Christine Buchmaier, Gregor Trimmel, Jenny Nelson, Saif A Haque

**Affiliations:** Department of Chemistry and Centre for Plastic Electronics, Imperial College LondonImperial College Road, London, SW7 2AZ, UK; Institute for Inorganic Chemistry, NAWI Graz, Graz University of TechnologyStremayrgasse 9, 8010, Graz, Austria; Institute for Chemistry and Technology of Materials, NAWI Graz, Graz University of TechnologyStremayrgasse 9, 8010, Graz, Austria; Department of Physics and Centre for Plastic Electronics, Imperial College LondonImperial College Road, London, SW7 2AZ, UK

**Keywords:** hybrid materials, organic electronics, photovoltaic devices, solar cells, spectroscopy

## Abstract

In this work, molecular tuning of metal xanthate precursors is shown to have a marked effect on the heterojunction morphology of hybrid poly(3-hexylthiophene-2,5-diyl) (P3HT)/CdS blends and, as a result, the photochemical processes and overall performance of in situ fabricated hybrid solar cells. A series of cadmium xanthate complexes is synthesized for use as in situ precursors to cadmium sulfide nanoparticles in hybrid P3HT/CdS solar cells. The formation of CdS domains is studied by simultaneous GIWAXS (grazing incidence wide-angle X-ray scattering) and GISAXS (grazing incidence small-angle X-ray scattering), revealing knowledge about crystal growth and the formation of different morphologies observed using TEM (transmission electron microscopy). These measurements show that there is a strong relationship between precursor structure and heterojunction nanomorphology. A combination of TAS (transient absorption spectroscopy) and photovoltaic device performance measurements is used to show the intricate balance required between charge photogeneration and percolated domains in order to effectively extract charges to maximize device power conversion efficiencies. This study presents a strong case for xanthate complexes as a useful route to designing optimal heterojunction morphologies for use in the emerging field of hybrid organic/inorganic solar cells, due to the fact that the nanomorphology can be tuned via careful design of these precursor materials.

## 1. Introduction

Interest in hybrid organic/inorganic blend photovoltaic technologies has increased markedly in recent years, with efficiencies now reaching 5.5%.[1] These technologies are desirable due to the promise of their ability to incorporate the attractive qualities of both materials, mainly the ability to solution process organic materials, and the superior mobility and chemical stability of inorganic materials. To date, hybrid devices have been fabricated using a wide range of inorganic materials, such as CdS,[2] CdSe,[3] Sb_2_S_3_,[4] PbS,[1,5] PbSe,[1] Bi_2_S_3_,[6] CuInS_2_[7] and ZnO[8] nanoparticles, along with many organic materials, such as conjugated polymers, low band gap polymers as well as small-molecular organic absorbers.[9] One of the major challenges with hybrid solar cells is the difficulty with processing both inorganic and organic materials in one step from the same solvent, due to the inherently different chemical and physical properties of both components. This problem is traditionally tackled by the use of organic capping ligands which allow for both components to be deposited together, but which also inhibit charge separation and inter-nanoparticle transport.[10] Therefore, typically ligand exchange processes are applied to reach high power conversion efficiencies (PCEs).[9a]

Recently, we developed an alternative method to the use of capping ligands by utilizing the thermal decomposition of a soluble precursor material to the inorganic nanoparticles, namely metal xanthate complexes.[11] Thin films comprising of a metal xanthate and polymer blend are spin coated from a common solvent and the metal sulfide phase is generated in situ with only gaseous by-products after a moderate (>120 °C) anneal in a nitrogen atmosphere. This method has been shown to give an increased charge generation yield and overall device performance compared to an equivalent device fabricated using the traditional ex situ method of blending nanoparticles with a polymer before thin film formation.[12] The in situ method is also potentially compatible with large-scale production, something that is seen as critical to the success of solution processed photovoltaic technologies[13] and the feasibility of preparing small modules on flexible substrates via the in situ route using metal xanthates as precursors and doctor blading as a coating method has already been demonstrated.[14]

Although there have been extensive studies on how to optimize the charge generation in these systems,[15] little is known about how to influence the nanomorphology of in situ prepared hybrid solar cells and the in situ formation process of the nanoparticles themselves. In analogy to polymer/fullerene solar cells, it is reasonable to assume that the nanomorphology of the absorber layer will be a key factor in further optimizing the PCEs of this emerging type of solar cells.[16] The crucial influence of the nanomorphology has been clearly shown for polymer/nanoparticle hybrid solar cells prepared via the classical approach using ligand-stabilized nanoparticles. Several reports demonstrated the effects of different nanoparticle shape (spherical,[3] rod-shaped)[17] or size[18] on device performance.

For in situ prepared hybrid solar cells, recent studies show that for different organic/inorganic material combinations quite different absorber layer morphologies are obtained. For example, using metal xanthates as precursors, comparatively big domain sizes have been observed for P3HT/CdS absorber layers,[11a] while well mixed phases were observed in PSiF-DBT/CIS layers.[7] The fact that the metal xanthates used for the preparation of the P3HT/CdS layers had a small ethyl ligand moiety, while for the in situ preparation of the CIS nanoparticles xanthates with longer and branched alkyl side chains were used, suggests that the precursor design has an influence on the nanomorphology formation. Moreover, it was observed that by increasing the ratio of inorganic to organic phase, the domain sizes of the inorganic material increases,[15,19] which leads, however, to the problem that due to the reduced amount of organic phase, a balanced charge transport of electrons and holes might be not provided. In terms of nanoparticle shape, besides spherical nanocrystals, elongated Bi_2_S_3_ nanorods have also already been obtained in a polymer matrix using metal xanthate precursors.[20]

**Figure 1:**
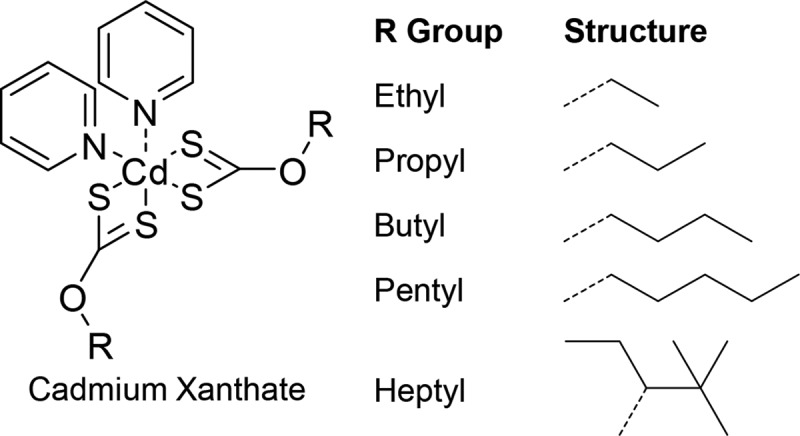
General chemical structure of the Cd-xanthate precursors used (bipyridine cadmium(II) dithiocarbonate) along with the different R Group moieties. It should also be noted that in the case of the heptyl xanthate, there are no pyridine adducts present, as due to the branched side chains, good solubility is already obtained without them.

Currently, no systematic studies on how the nanomorphologies of in situ prepared absorber layers can be tailored in order to optimize the device performance exist. Therefore, for this study, we have chosen the material combination P3HT/CdS as a model system to investigate if the absorber layer nanomorphology can be tuned by the molecular design of the precursors. All previous studies on this material have focussed only on xanthate molecules with one common ligand moiety. In this study we not only attempt to gain an understanding on how the metal chalcogenide nanocrystals grow within the polymer matrix and how the nanomorphologies are formed, we also investigate the effect that small changes in the solubilizing alkyl groups of the precursors have on the nanocrystal growth and nanomorphology formation, as well as the photophysics of hybrid photoactive layers. To this end, we investigate five cadmium xanthates with different alkyl moieties, namely, ethyl, propyl, butyl, pentyl and 2,2-dimethylpent-3-yl, which is referred to as heptyl in the further course of the study (see **Figure** **1**).

Blends of five xanthate precursor materials (see Figure 1) and P3HT were spin coated and annealed at 160 °C in a nitrogen atmosphere to form the hybrid polymer/inorganic networks. These films were first investigated using TEM showing markedly different nanomorphologies. Moreover, the growth process of each system was then studied in detail using simultaneous GIWAXS (grazing incidence wide-angle X-ray scattering) and GISAXS (grazing incidence small-angle X-ray scattering) during the annealing process to monitor the crystal growth and gain a better understanding of the formation of the different morphologies observed by TEM. Furthermore, the thin films were subjected to photophysical characterisation, including UV–VIS absorption, photoluminescence, fs-TAS (femto-second transient absorption spectroscopy) and μs-TAS (micro-second transient absorption spectroscopy) to observe which effects the different morphologies have on the photophysical processes, including polymer exciton lifetimes and the relative yields and lifetimes of free charge carriers, within the device active layers. Generally, it was observed that an increase in mixing resulted in a decrease in exciton lifetime and an increase in the relative yield of charge carriers. Finally series of complete devices were fabricated using an inverted device architecture of ITO/TiO_2_(dense)/CdS/P3HT:CdS(blend)/MoO_3_/Ag to measure the overall effect of ligand moiety on photovoltaic performance. These devices showed that the optimum nanomorphology for charge generation, i.e., good mixing, did not correlate with the optimum device performance and highlight the importance of being able to finetune hybrid heterojunctions at the nanoscale. Brief descriptions of all techniques can be found in the experimental section and full details can be found in the Supporting Information.

## 2. Results and Discussion

**Figure 2:**
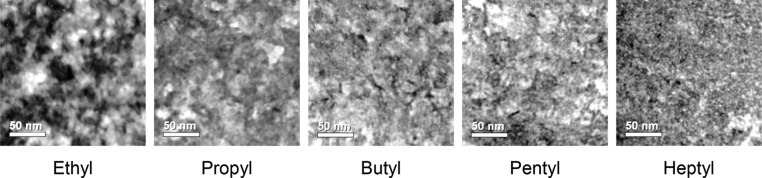
Bright field TEM (transmission electron microscopy) images of thin films of hybrid P3HT/CdS heterojunctions, fabricated from a series of Cd-xanthate precursors with increasing ligand moieties from left to right. The darker regions of contrast are indicative of a higher concentration of cadmium.

The morphology of in situ formed P3HT/CdS hybrid blend thin films was first studied using bright field TEM, typical images of which can be seen in **Figure** **2**. From left to right the images show films fabricated using a xanthate precursor of increasing ligand moiety size, from ethyl through to heptyl. It is apparent that as the ligand moiety is changed the morphology of the blends changes, with the domain sizes within the films becoming smaller as a larger ligand is used. Upon close inspection of the TEM image of the heptyl sample (Figure 2, Heptyl), discrete particles can be seen. These particles, although still observable, become less easy to isolate visually when moving towards a smaller ligand moiety on the left hand side of the figure. It appears that an increase of the alkyl chain size of the precursors results in a reduction in the aggregation of the cadmium sulfide nanoparticles upon decomposition of the xanthate precursors. It is worth noting however that the TEM images only provide a top-down look at the morphology and although we can see a trend in this direction we can only assume that the landscape looks similar in the z-direction as well. The significantly different morphologies of the P3HT/CdS hybrid films as shown in the TEM images in Figure 2 support our hypothesis that molecular precursor design is a valuable tool for tuning the nanomorphology of in situ prepared polymer/nanocrystal absorber layers towards efficient charge generation and transport. However, target-oriented molecular design of the precursors requires knowledge of the processes going on during the in situ formation of these films.

The potential reasons for the development of different nanomorphologies are numerous and could be, for example, influenced by differences in solubility or rather different miscibility with the used polymer P3HT, by different phase separation or crystallization behavior of the metal xanthates in the precursor film, by decomposition of the xanthates and subsequent nanocrystal growth at different temperatures, or by different growth kinetics of the nanocrystals. To understand the processes during the formation of the nanocomposite layers and to gain knowledge on which parameters are most critically influencing the formation of the different nanomorphologies, the thermal annealing step was thoroughly investigated on the nanometer scale. This was done, in particular, by time resolved GIWAXS as well as GISAXS measurements using synchrotron radiation.

**Figure 3:**
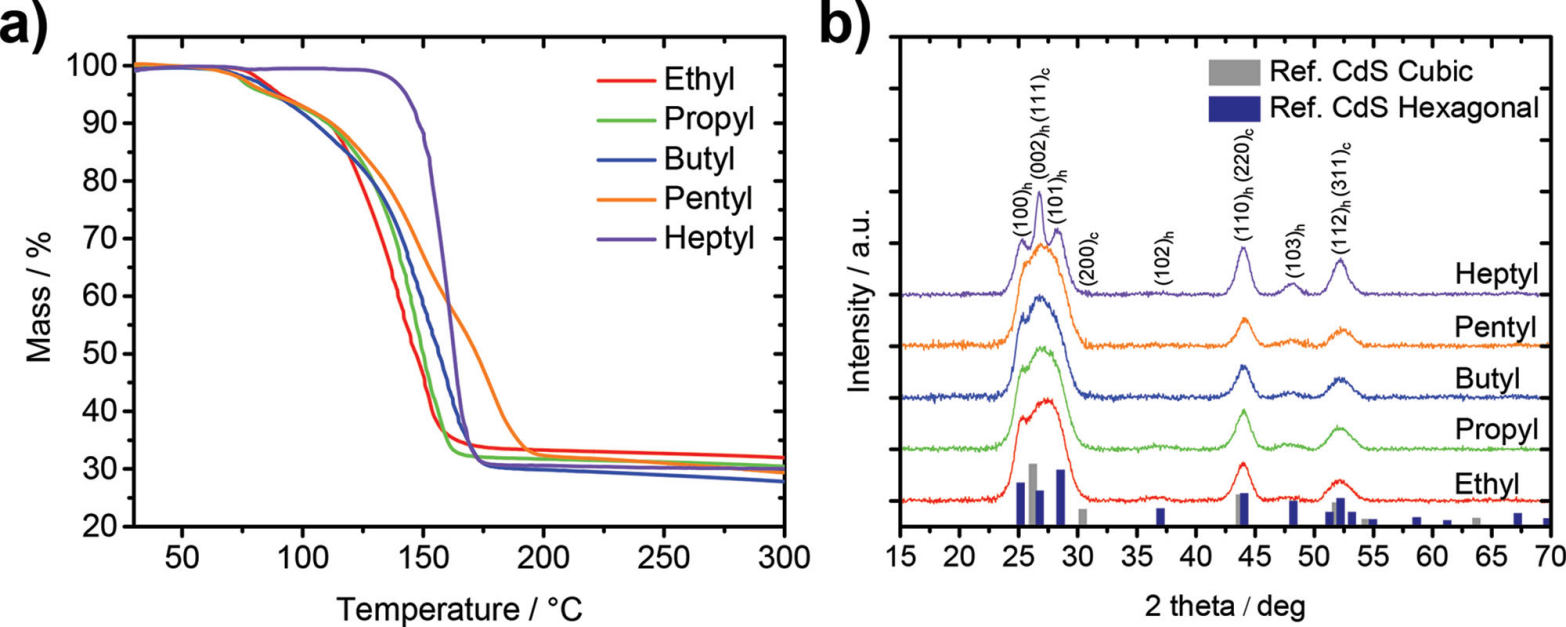
a) Thermogravimetric analysis of the thermal decomposition of the Cd-xanthates (ethyl, propyl, butyl, pentyl, heptyl) investigated in this study. b) X-ray diffraction patterns of P3HT/CdS nanocomposite layers prepared from the different Cd-xanthates via thermal annealing at 160 °C for 60 min. The main peaks of both the hexagonal as well as the cubic phase are labelled with their Miller indices.

To get a first indication about possible differences in the thermal decomposition of the different xanthates, thermogravimetric analysis (TGA) was carried out. The results presented in **Figure** **3**a show that the decomposition of ethyl, propyl, butyl and pentyl xanthates proceeds via a two-step scheme. The first decomposition step starts at approx. 70 °C and the second at about 120–130 °C. While the decomposition characteristics in the first step are similar in these samples, the second step is quite different. After a mass loss of about 18%, the ethyl xanthate has an onset of a steep mass loss at 122 °C while the phase of steep mass loss is shifted to slightly higher temperatures for propyl (125 °C), butyl (126 °C) and pentyl (130 °C) xanthates. Moreover, the slope of the mass loss in the second decomposition step decreases going to higher chain length of the xanthates’ ligand moiety. The heptyl xanthate shows a markedly different behavior. It is thermally stable up to a temperature of 135 °C and decomposes comparably fast in one step. This is possibly caused by the fact that in the heptyl xanthate no pyridine ligands, which lower the decomposition temperature of metal xanthates,[21] are present, or could be due to the decreased number of beta hydrogens available, which facilitate the Chugaev elimination. The mass loss during the conversion of the respective Cd-xanthates to CdS is between 68 and 72%, which matches well with the theoretical values taking the small uncertainties of the used measuring setup into account. Moreover, the Cd-xanthate-bipyridyl compounds lose about 5% less weight than theoretically expected. This might be due to incomplete complexing of all Cd-xanthate molecules with two pyridines.

These distinct differences in the thermal decomposition behavior of the investigated metal xanthates suggest possible differences in the formation/crystallization of the CdS nanocrystals in the P3HT matrix. X-ray diffraction measurements of the P3HT/CdS nanocomposite layers prepared from the different Cd-xanthates after the thermal annealing step were performed to detect a possible variation of the crystal structure or the primary crystallite size of the CdS nanocrystals in the final stage after the heating process. Figure 3b shows the respective X-ray diffraction patterns and reference patterns for hexagonal CdS (PDF 41–1049) as well as cubic CdS (PDF 01–080–0019). The main peaks of both crystal structures are labelled with their Miller indices followed by the suffix h or c to indicate the hexagonal (wurtzite) or cubic (sphalerite) phase, respectively. Comparing the measured patterns with the reference patterns, it is apparent that the nanocrystals exhibit a blend of hexagonal and cubic crystal structure, which stems from mixed cubic and hexagonal stacking. This mixed layer stacking is not unusual for CdS nanocrystals and was already observed in other studies on CdS[22] as well as isostructural ZnS nanocrystals.[23] The relative amounts of cubic and hexagonal crystal phase in the samples are hard to determine due to the broadening of the peaks, however, the distribution ratio seems to be comparable in the ethyl, propyl, butyl, and pentyl samples. In the heptyl sample, the (100)_h_ (002)_h_ and (101)_h_ peaks are more distinct, indicating that the overlapping (111)_c_ peak is less intense than in the other samples, which lets us conclude that the amount of hexagonal phase might be higher in the heptyl sample. The fact that the middle peak (002)_h_ is much more intense than the neighboring two indicates that there is also some cubic crystal structure present, as this peak is superimposed by the (111)_c_ peak of the cubic crystal structure. Moreover, in all samples the (200)_c_ peak is hardly visible in the diffraction patterns and also the (102)_h_ peak is not as pronounced as the (103)_h_ peak, which matches findings of a previous study investigating mixed hexagonal and cubic stacking.[23]

The better separation of the three peaks of the hexagonal phase in the heptyl sample could also originate from a bigger primary crystallite size in this sample, however, according to an estimation of the primary crystallite size using the Scherrer formula, the primary crystallite size in the heptyl sample is not significantly bigger than in the other samples. The broadening of the peaks for the estimation of the primary crystallite size via the Scherrer formula was determined by fitting the (110)_h_ (220)_c_ peak at approx. 44° 2 theta with the two individual peaks stemming from the hexagonal and cubic phase. For all samples, the primary crystallite size is approximately 7 ± 1 nm, which is in line with the size of the nanocrystals found in the TEM images. Thus, different crystallite sizes can be excluded as a possible factor leading to the different nanomorphologies of the hybrid absorber layers and it appears that the different morphologies originate from a different degree of agglomeration of the single nanocrystals.

**Figure 4:**
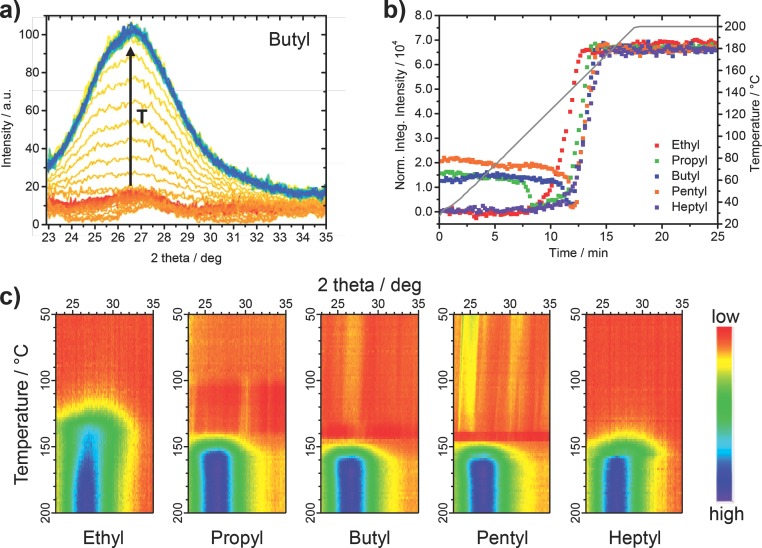
a) GIWAXS patterns of a P3HT/Cd-butyl xanthate film measured between 22.9 and 35° 2 theta during a heating run from room temperature to 200 °C with a heating rate of 10 °C min^−1^ showing the evolution of the most intense peak of CdS at around 27° 2 theta. The increasing temperature (T) is indicated with a black arrow in the graph. b) Normalized integrated intensity of the GIWAXS patterns of the different P3HT/metal xanthate samples calculated between 22.9 and 35° 2 theta plotted versus reaction time and temperature. The corresponding GIWAXS patterns are shown in Figure S1 in the Supporting Information. c) Comparison of the temperature-dependent evolution of the most intense CdS peak during the heating run.

Next, time resolved GIWAXS measurements were performed to obtain insight into the formation of the CdS nanocrystals in the P3HT matrix. The use of synchrotron radiation and the grazing incidence setup enabled us to probe directly approx. 150 nm thick films, comparable to those used in devices, at a time resolution of 11 s. These short intervals are necessary, as it is known from former experiments that the formation of the metal sulfides from metal xanthates occurs in a quite fast reaction step.[24] Due to a limited 2 theta range of the detector used, we focused on the formation of the broad and most intense peak of CdS in the samples (superimposition of the (110)_h_, (002)_h_, (101)_h_ and (111)_c_ reflections) which is situated around 27° 2 theta, and chose to monitor the range between 22.9 and 35° 2 theta. The temperature-dependent changes in the GIWAXS patterns during a heating run from room temperature to 200 °C with a heating rate of 10 °C/min are shown for the P3HT/Cd-butyl xanthate sample in **Figure** **4**a. The GIWAXS patterns of all investigated samples are summarized in Figure S1 in the Supporting Information. In Figure 4a, the evolution of the most intense peak of CdS can be clearly seen. The shape of the peak is slightly different to the one observed in the X-ray diffraction measurements shown in Figure 3b. This is due to peak broadening caused by the grazing incidence setup and also because of the fact that for the X-ray diffraction measurements much thicker films were used. However, also in the GIWAXS pattern, a shoulder at about 25.5° 2 theta similar to, but not as pronounced as in the X-ray diffraction pattern is observed. Moreover, in some experiments, as it can be seen also in Figure 4a, already at room temperature some weak peaks (around 23, 27, 33 and 34.5° 2 theta) are visible, which may stem from partial crystallizing of the Cd-xanthate in the precursor layer. These peaks from the precursor material are present in the sample until a certain temperature where they vanish shortly before the CdS nanocrystals are formed. This behavior is more visible in Figure 4b. Here, the normalized integrated intensity of the GIWAXS patterns of the different P3HT/metal xanthate samples calculated between 22.9 and 35° 2 theta are plotted versus reaction time along with the temperature ramp in grey. The vanishing of the peaks of the precursor material and the subsequent formation of the CdS nanocrystals can be also seen in Figure 4c, in which the temperature-dependent changes in the diffraction pattern during the heating run are visualized in a surface plot.

The surface plots illustrate that the ethyl and heptyl xanthates do not have peaks at room temperature, while in the propyl, butyl, and pentyl samples some of these previously discussed weak peaks are visible. In the pentyl xanthate sample these peaks are most significant. It is also apparent that these “precursor-peaks” disappear at different temperatures. The integrated intensity of the diffraction patterns starts to decrease in the propyl sample already slightly above 100 °C while it stays stable until about 130 °C in the butyl sample and until 135 °C in the pentyl sample. In the ethyl and heptyl sample no “precursor-peaks” are observed and in the integrated intensity only the formation of the CdS nanocrystals can be detected. The nanocrystal formation starts already at 125 °C in the ethyl sample, which is approximately 5–10 °C higher than the onset temperature of the fast second decomposition phase, as determined by thermogravimetric measurements (see Figure 3a). In the other samples, the onset of the nanocrystal growth is shifted to slightly higher temperatures (140 °C for the propyl and heptyl samples and 145 °C for the butyl and pentyl samples). The fact that peaks of the precursor material are observed in the X-ray diffraction patterns of the propyl, butyl and pentyl samples suggests that the agglomeration of the nanocrystals after the heating step is influenced by a phase separation of polymer and metal xanthate phase already in the precursor layer. However, for the ethyl sample no peaks of the precursors were found, which is surprising because the ethyl sample exhibits the largest domain sizes. It is possible that also in this sample the polymer and the metal xanthates phase separate, though the precursor molecules do not form crystalline domains and thus, no peaks are visible in the X-ray diffraction pattern. The observation that in the heptyl sample the polymer and nanoparticle phases are well mixed (see TEM images in Figure 2), lets us conclude that the solubility and the mixing with the polymer of the heptyl xanthate, due to the branched alkyl chains, is very high and as a result no pronounced phase separation occurs in the heptyl sample.

It is interesting that the nanocrystal growth is much slower in the ethyl sample compared to the others. This can be seen in the surface plots in Figure 4c and as well in the 3D-plots in Figure S2 in the Supporting Information. The formation of the main peak of CdS in the sample at around 27° 2 theta is quite smeared over a larger temperature range, which is a significant difference compared to the other samples, where fast and abrupt nanocrystal growth is observed. After this short nanocrystal growth phase, the intensity of the peak as well as the shape and width of the peak stay constant indicating no further nanocrystal growth in this last phase of the heating run.

**Figure 5:**
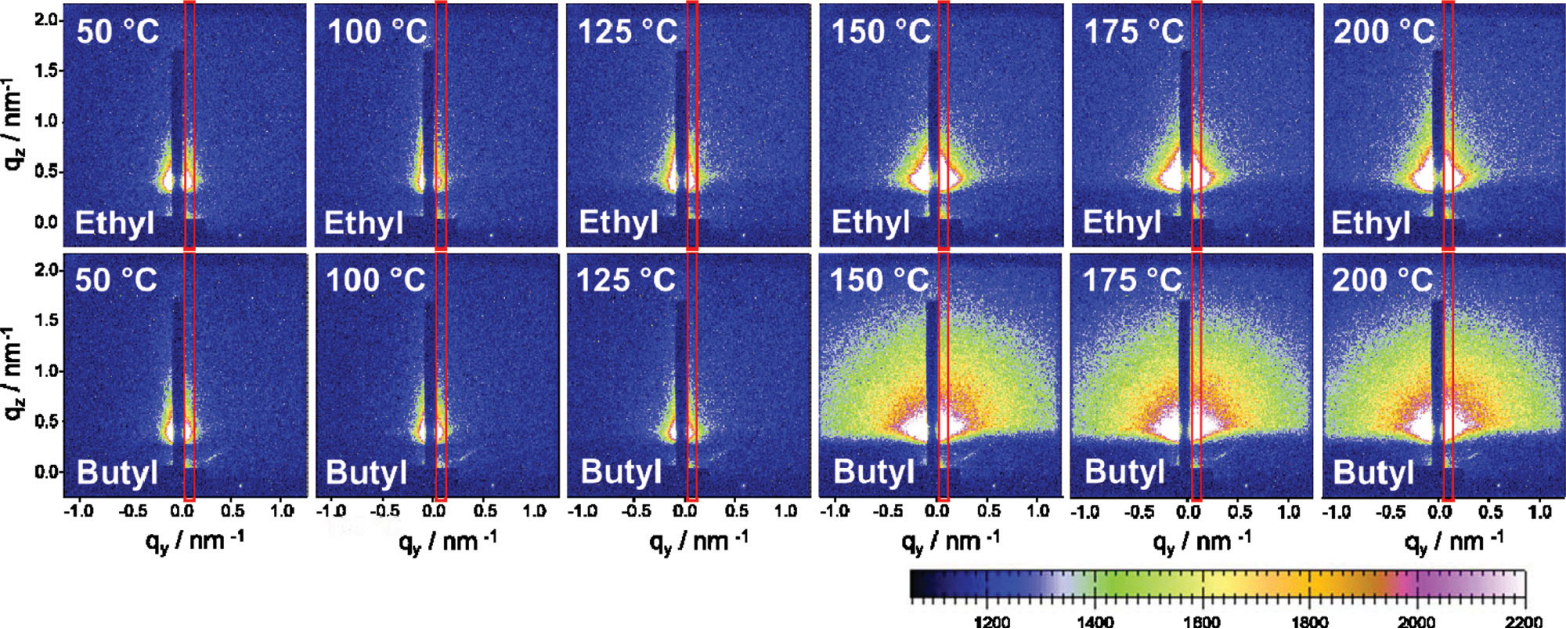
GISAXS images of the ethyl and butyl xanthate samples at different temperatures during the heating run from room temperature to 200 °C. The red boxes indicate the vertical areas used for integration.

**Figure 6:**
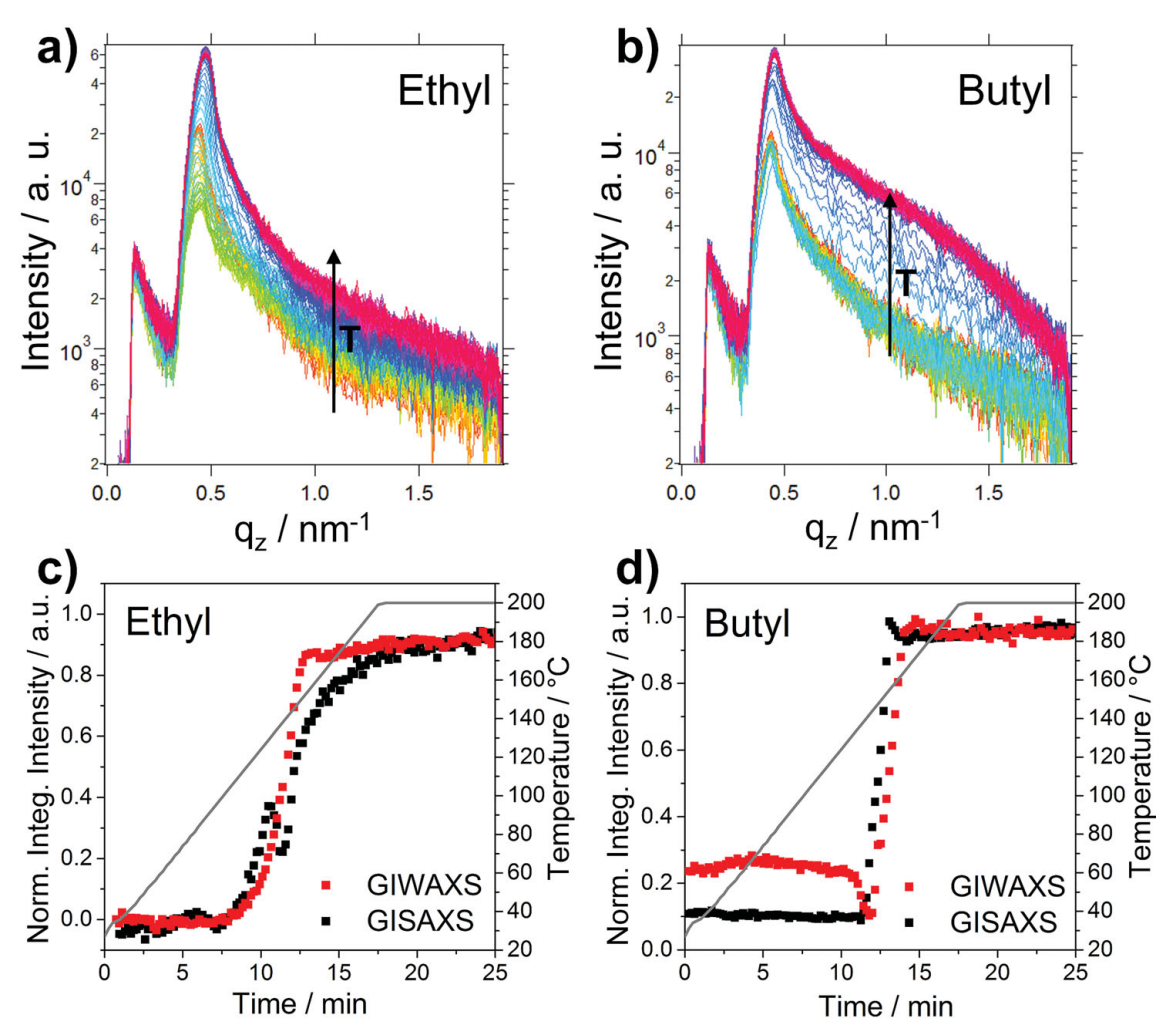
Temperature-dependent evolution of the vertical cuts of the GISAXS patterns of a) the ethyl-sample and b) the butyl-sample. The increasing temperature (T) is indicated with a black arrow in the graphs. c, d) Comparison of the evolution of the integrated intensities of the GIWAXS and GISAXS signals during the heating run.

Time-resolved GISAXS measurements were performed simultaneously with the GIWAXS measurements. The GISAXS patterns of the ethyl xanthate and the butyl xanthate at different temperatures during the heating run are shown in **Figure** **5**. The areas for vertical integration are indicated with a red box in the GISAXS images and the resulting vertical cuts at *q*_y_ = 0.058 nm^−1^ are presented in **Figure** **6**. The GISAXS images as well as the vertical cuts of the full set of samples are summarized in Figure S3 and S4 in the Supporting Information.

The integrated intensities extracted from the GISAXS data reveal structural changes in the layers directly before the growth of the nanocrystals starts. These structural changes have their origin in the decomposition of the cadmium xanthates and evaporation of volatile decomposition products out of the layer, which leads to a compaction of the layer and an overall increase in electron density. This increase of electron density is also reflected in the shift of the Yoneda peak (*q*_z_ ≈ 0.45 nm^−1^) towards higher q-values during the heating run. The idea that the structural changes are stemming from the decomposition of the precursors is also supported by the fact that the observed increase of the integrated intensity of the GISAXS signal occurs in the same phase of the heating run, where the peaks of the precursors vanish in the GIWAXS pattern, as it can be seen in Figure 6d.

Moreover, the GISAXS measurements suggest that there is an agglomeration of the nanocrystals after the growth phase in the ethyl sample, which is indicated by a further increase of the integrated intensity of the GISAXS signal after the nanoparticle growth (see comparison of the integrated intensities of GISAXS and GIWAXS signals in Figure 6c). This might be a reason for the formation of markedly bigger domain sizes in the ethyl sample. In all the other samples, no particle aggregation after the nanoparticle growth is observed (see Figure S4, Supporting Information), which lets us assume that the nanoparticle agglomerates found in the propyl, butyl and pentyl samples are mainly already determined by the phase separation of polymer and cadmium xanthates in the precursor layer as discussed before in the GIWAXS section.

After gaining insight into the growth of CdS via the decomposition of the different xanthate precursors, we turned the focus of our work to study the effect that the different precursor materials and their respectively different morphologies had on the photophysical properties of these hybrid heterojunctions. Spectroscopic studies were performed on thin films prepared in an identical manner to those previously studied in the GIWAXS and GISAXS measurements and investigated using TEM.

**Figure 7:**
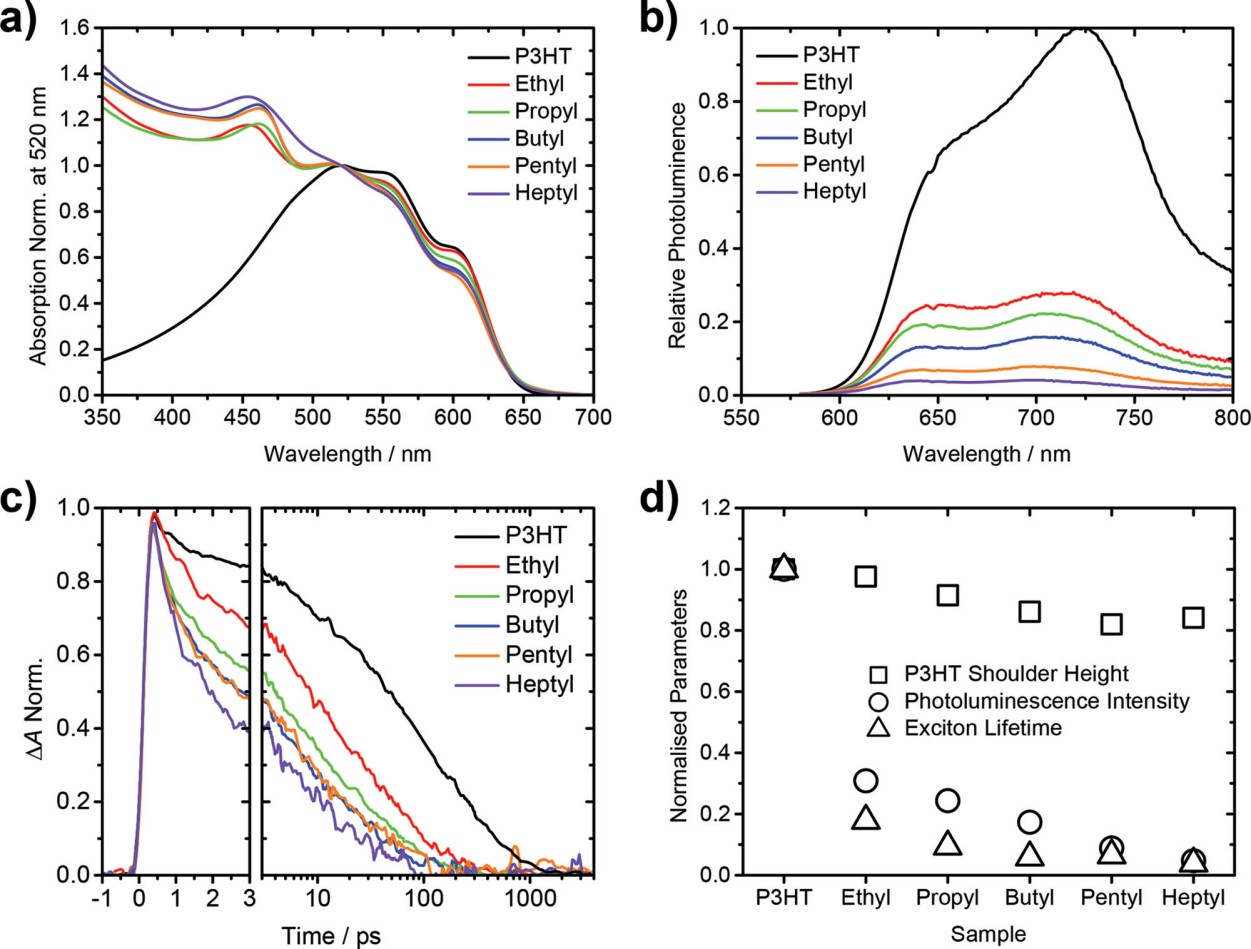
a) Steady-state absorption measurements of P3HT/CdS heterojunctions normalized at 520 nm. b) Photoluminescence quenching measurements relative to a neat P3HT sample (excited at 567 nm). c) Femtosecond transient absorption (fs-TAS) decays of samples measured at 1270 nm, corresponding to the P3HT exciton absorption. d) Normalized parameters from each of the other figures showing the trends observed from each of the different techniques.

Considering first the steady-state absorption of these films, in **Figure** **7**a it can be seen that the CdS nanoparticles absorb light in a complementary region of the spectrum compared to P3HT, highlighting one of the potential advantages that a hybrid system of this type offers. The spectra have been normalized at 520 nm and plotted against a measurement of neat P3HT. It is clear that there is already a distinct trend to accompany the observation of the different morphologies. In the absorption spectrum of P3HT (shown in black) a distinct shoulder is seen at ≈600 nm, a feature of the P3HT spectrum that is attributed to the degree of aggregation between adjacent polymer chains.[25] The relative size of this shoulder compared to the P3HT maximum absorption is a good indication of the level of aggregation of P3HT, or, in other words, is an indication of crystalline P3HT domain size. In Figure 7a, it can be seen that as the Cd-xanthate ligand moiety length is increased, the relative height of this shoulder decreases. This can be seen more clearly in Figure 7d, where the squares represent the relative absorption at 600 nm compared to the normalized absorption at 520 nm. The decrease in shoulder height indicates that as a larger ligand moiety is used, the aggregation of the P3HT decreases. This correlates also with XRD measurements (see Figure S5 in the Supporting Information), which show a general trend of decreasing P3HT crystallinity with increasing ligand moiety length used. This observation furthermore supports the TEM images where the P3HT domain size is visibly seen to decrease as a larger ligand moiety is used (Figure 2).

The nanomorphology of these photoactive layers was then probed by using the photophysical processes within these heterojunctions as an indicator. The approximate domain size of P3HT was investigated by studying the steady-state photoluminescence (PL) of these films. Since the same polymer M_n_ and regioregularity was used for all samples, exciton quenching by CdS is believed to be the only cause of a reduced PL intensity. The P3HT was solely excited at a wavelength of 567 nm, the results of which can be seen in Figure 7b. There are two key observations to be made from these measurements as a longer ligand moiety is used: 1) the amount of emission from the P3HT decreases and 2) the emission is blue shifted. The emission data is summarized in Figure 7d, with the integrated emission relative to neat P3HT shown in circles. An increase in exciton quenching is a typical finding which indicates a reduction in the relative domain size of a polymer blended with an acceptor material and in this system it appears that as a longer ligand moiety is used an increase in quenching is observed.

We can attribute this to the P3HT excitons having, on average, a shorter distance to travel to a CdS interface, resulting in more excitons being quenched before they radiatively decay. The blue shifting in the spectral maximum is another indication of a reduction in aggregation of the P3HT, something that is observed when comparing the polymer emission in a dilute solution, where aggregation is at a minimum, and in thin film.[26] This has also been observed when the packing density of P3HT has been tuned through controlling the regioregularity.[27]

The decay kinetics of the P3HT excitons were studied using femtosecond transient absorption spectroscopy (fs-TAS). With this method both the photoinduced absorption of P3HT excitons and polarons can be observed.[28] We focus here on the kinetics of the P3HT excitons, which are the only absorbing species at 1270 nm.[29] Figure 7c shows the kinetics at a probe wavelength of 1270 nm following excitation with a 570 nm laser pulse with an intensity of 3.9 μJ cm^−2^. Relative lifetimes of these signals were calculated from determining the times at which the signals dropped to 1/e of the original amplitude and are shown as triangles in Figure 7d. It can be observed that as a larger ligand moiety is used the lifetime of the exciton is reduced. As with the other two steady-state techniques, this indicates that the domain size of the P3HT is decreasing and is in correlation with the TEM results (Figure 2).

Although these three techniques, together with the previous results, present a strong case for the larger ligand moiety having the effect of decreasing the domain size of P3HT, these measurements alone do not directly probe the charge generation within these hybrid heterojunctions. It is still possible that the larger leaving groups could increasingly disrupt the P3HT packing when leaving the film and lead to the previous observations. To rule out this possibility and to get further information on the effectiveness of these heterojunctions at generating charges, μs-TAS (micro-second transient absorption spectroscopy) was employed to measure both the relative number of free charges generated in these 5 different systems and also the lifetime of these transient species. It is noteworthy that fs-TAS can also be used to probe the yields and dynamics of polarons as well as excitons, however on the timescales measured there is still some overlap in signal between P3HT excitons and polarons at the characteristic polaron wavelength.[29] For this reason and the fact that μs-TAS has been extensively used previously to study this system[11,15] and others similar[28,30] it was chosen instead. Details of this particular setup are available in the Supporting Information.

**Figure 8:**
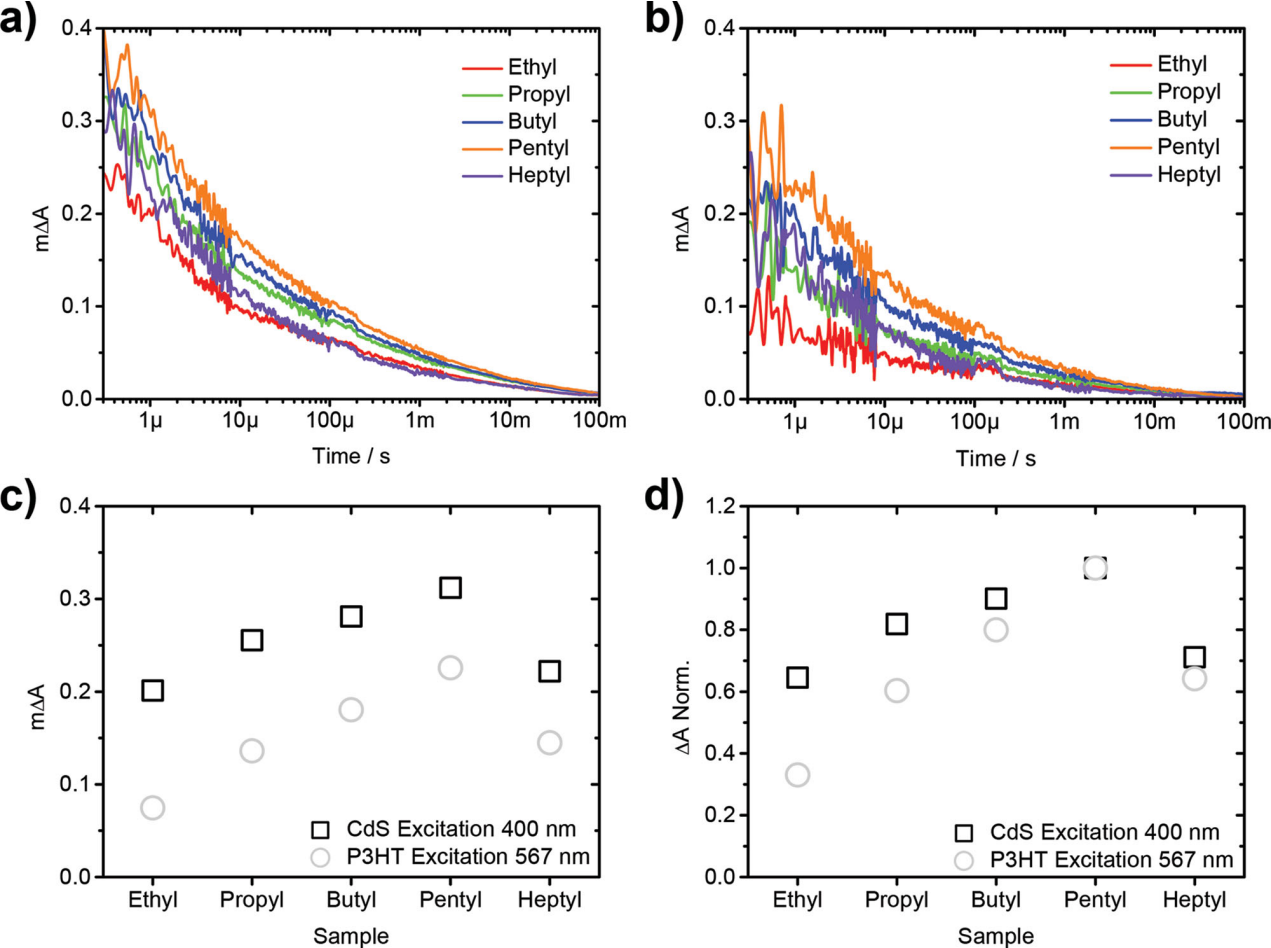
Transient absorption spectroscopy (TAS) decays at a) 980 nm using an excitation wavelength of 400 nm exciting predominantly CdS domains and b) 567 nm exciting exclusively P3HT domains. c) The amplitudes of all signals at both wavelengths with d) the data normalized to the highest signal.

The relatively low spectral overlap in the absorption of P3HT and CdS allows for each component to be excited reasonably selectively. Consequently, both the electron transfer reaction from P3HT to CdS and the converse hole transfer reaction from CdS to P3HT were separately studied. The P3HT was excited using a pump wavelength of 567 nm, where there is no absorption by the CdS, and the CdS was excited at 400 nm where there is only a small absorption contribution from the P3HT. In both cases the signals are scaled for the number of pump photons absorbed to account for small variations in film thickness between samples and also the difference in absorbance between samples at 400 and 567 nm. Both regions were pumped with the same approximate number of photons and were probed at a wavelength of 980 nm, which has previously been shown to correlate to the P3HT hole polaron.[6,11] The transient traces are presented in **Figure** **8**a and b following the decay of the P3HT hole polaron but as a visual aid the amplitudes at 1 μs are plotted in Figure 8c and the amplitudes normalized to the maximum amplitude are shown in Figure 8d.

In Figure 8a and b the decays of all samples show the transient species to be relatively long lived, with all traces having a t_50%_ (mΔA at 300 ns) of over 10 μs. There is also an observable difference in the amplitudes of all samples in both the CdS excitation and P3HT excitation case. It can be seen that in all cases there is a higher efficiency of charge generation when exciting the CdS in comparison to when exciting the P3HT, or, in other words, the hole transfer reaction is working more effectively than the electron transfer. This is something that has previously been observed in the P3HT/CdS system prepared using ethyl-xanthates, where the charge generation yields were studied thoroughly for a range of compositions of this heterojunction.[15b] The second observation is that, in both excitation cases, as a longer ligand moiety is used, the amount of charges generated steadily increases until the case of heptyl, where the yield is slightly reduced. This increase in charge generation yield can be explained by an increased mixing, as a more intimately mixed system results in excitons being generated closer to an interface and thus being more likely to separate into free charges before the exciton relaxes. This finding supports the observations in both the steady-state photoluminescence and fs-TAS exciton lifetime measurements of Figure 7. We can tentatively explain the reduction in yield for the heptyl case as being due to the absence of agglomeration of the nanocrystals, which leads to domain sizes becoming so small that either, there is a fast recombination pathway that is quicker than the measurement timescale, resulting in the observed signal being lower than expected by 1 µs, or, the reduced ability of the polaron to delocalize results in a lower driving energy for separation. This is consistent with the findings of Dayal et al.[31] who observed, using microwave conductivity measurements, that increasing the aspect ratio of QDs to rods and tetrapods resulted in an increase in charge generation. An alternative explanation could be that the increased amount of wurtzite relative to cubic CdS in these samples, as observed in the XRD (Figure 3b), could potentially change the polymer/nanoparticle interface and affect the amount of geminate recombination losses between bound polaron pairs.

When comparing the relative effect of changing ligand length on charge generation initiated by hole transfer compared to electron transfer, it can be seen that the effect is relatively small here compared to the more significant effect on the electron transfer. This can be most clearly seen in Figure 8d, where the data has been normalized. In this figure, it can be seen that when exciting the CdS phase, the difference between the ethyl and pentyl sample is an increase of approximately 50% whereas when exciting the P3HT there is an increase of 200% between the same two systems. This can be explained due to the difference in the nature of the species formed after excitation in CdS compared with in P3HT. Upon photon absorption in P3HT it is well documented that bound excitons are formed and they have a low diffusion length of <10 nm.[32] The lifetime of these excitons is indeed short-lived and is what was measured previously, using fs-TAS shown in Figure 8c. It is therefore expected that a finer mixing of both components will result in an increase in charge generation due to more P3HT domain sizes being reduced to a length scale of nearer the diffusion length.

The reason for this increase in mixing having less of an effect when photons are absorbed by the CdS is because, due to the higher dielectric constant, a bound exciton is not expected to be formed and instead free charges are likely to be present in the inorganic layer without the need to diffuse to an interface. These charges in CdS have been shown to have a much longer lifetime than the excitons in P3HT[29] allowing for longer diffusion pathways to an interface. We have also measured hole transfer from antimony sulfide to P3HT to occur over extremely long timescales.[33] This observation of hole transfer showing very little dependence on morphology in comparison to electron transfer was previously seen in this system whereby the hole transfer was found to be not very dependent on the composition of blends, whereas after P3HT excitation the amount of charges yielded was highly dependent on having a high amount of acceptor material.[15b] It is also expected that this observation is actually even more heavily pronounced than what is measured here as there is still a small amount of light absorption by the P3HT at 400 nm and it could be charges generated from absorption here that accounts for the small dependence on ligand moiety size.

Finally, a series of complete devices was fabricated to test the overall effect of tuning the morphology through ligand moiety design. Devices were fabricated of the structure ITO/TiO_2_(dense)/CdS/P3HT:CdS(blend)/MoO_3_/Ag, with the P3HT:CdS layer being identically fabricated to the films used throughout the study. Devices were tested using an AM 1.5 source, details of which can be found in the experimental section.

**Figure 9:**
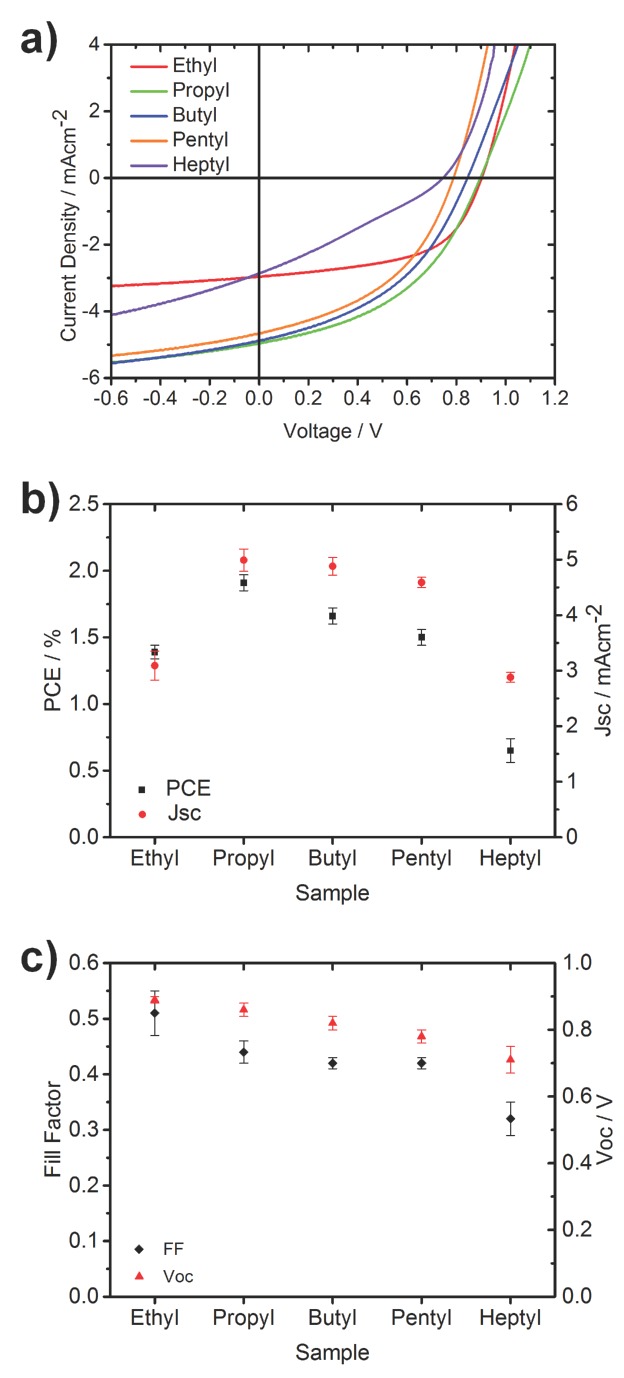
a) Current–voltage characteristics of typical devices, b) average power conversion efficiencies (PCE) and short circuit currents (Jsc) of an average of the 10 devices with the highest PCE, c) average fill factors (FF) and open circuit voltages (*V*_oc_) of the same 10 most efficient devices.

In **Figure 9**a the current voltage characteristics are presented for representative devices for each ligand used and in Figure 9b and c the average characteristic device parameters are shown for the top 10 devices in terms of PCE. This data is also summarized in **Table 1**. In Figure 9b we can see that the variation in device performance is mainly governed by the short circuit current, with the devices made with the propyl ligand showing both the highest current and efficiency. All other devices can be ordered by their short circuit current and power conversion efficiency and be seen to have the same trend. It is pertinent to note, however, that the trend in the short circuit currents does not match the trend previously observed in the charge generation yield, as measured by μs-TAS, whereby the films made with the pentyl ligand were found to generate the highest number of charges. A correlation between charge generation yield and short circuit current is one that has been seen in several all organic systems,[34] but is not what has been observed previously in this system. We recently reported on the mismatch between the optimum blend ratio for charge generation and the optimum for efficiency in complete devices.[15b] This was attributed to the fact that although a higher heterojunction surface area could be created by increasing the loading of the inorganic component within the polymer and as a result increase the charge photogeneration efficiency, at a certain loading this became so high that the domain size of P3HT was reduced to a point where the charges were not effectively extracted. A similar behavior was found for polythiophene/ZnO nanoparticle hybrid solar cells, where a finer mixed morphology led to higher charge separation yields, however, not to increased short circuit currents due to a limited hole mobility in the polymer phase because of the smaller domain sizes.[8] It is likely that an analogous problem is being observed here whereby at a given blend ratio the morphology has an optimum point where the balance between interface area and domain connectivity is found. In this system, it appears that this sweet-spot is found with the propyl ligand. In Figure 9c it can be seen that both the fill factor and open circuit voltage are reduced when a metal xanthate with a longer ligand is used. This is likely due to the finer mixing resulting in an increase in the non-geminate recombination.

**Table 1:** Average values for *J*_sc_, *V*_oc_, FF, and PCE of the same top 10 devices plotted in Figure 9

Sample	*J*_sc_ [mA cm^−2^]	*V*_oc_ [V]	FF	PCE [%]
Ethyl	3.09 ± 0.26	0.89 ± 0.01	0.51 ± 0.04	1.39 ± 0.05
Propyl	4.99 ± 0.20	0.86 ± 0.02	0.44 ± 0.02	1.91 ± 0.06
Butyl	4.88 ± 0.16	0.82 ± 0.02	0.42 ± 0.01	1.66 ± 0.06
Pentyl	4.59 ± 0.09	0.78 ± 0.02	0.42 ± 0.01	1.50 ± 0.06
Heptyl	2.88 ± 0.09	0.71 ± 0.04	0.32 ± 0.03	0.65 ± 0.09

## 3. Conclusion

In summary, this study shows how the nanomorphology of in situ prepared hybrid solar cells can be tuned efficiently by the design of the molecular nanocrystal precursors. Small changes in the structure of the used metal xanthates induce significant changes in the obtained absorber layer nanomorphologies. Longer alkyl chains of the xanthate moieties lead to a better mixing of the polymer and nanoparticle phase, to smaller domain sizes and a lower crystallinity of the polymer phase in the P3HT/CdS absorber layers. The formation of these different morphologies is determined by agglomeration of the nanocrystals, which have a similar size in all the investigated samples. As suggested by time resolved X-ray scattering measurements, the agglomeration of the nanocrystals is influenced by growth temperature and kinetics of the nanocrystals as well as phase separation of polymer and metal xanthate phase already in the precursor layer.

The different nanomorphologies strongly influence the optoelectronic properties of the absorber layers and in turn also the performance of the hybrid solar cells and a complex interplay between these parameters is disclosed by the performed characterizations. The highest level of quenching of the photoluminescence of the polymer phase by the CdS nanocrystals was found for the heptyl sample, indicating that the heptyl sample was the most intimately mixed. This was also supported by fs-TAS showing the shortest P3HT exciton lifetime in this system. However, optimum charge generation, probed by μs-TAS, was observed in the pentyl sample showing that in the heptyl sample, due to non-agglomerated nanocrystals, the domain sizes are most presumably too small for the efficient generation of long-lived charges, which is facilitated by bigger domain sizes. Overall, the best power conversion efficiencies were obtained with hybrid solar cells prepared using propyl xanthates. This originates from the fact that besides efficient charge generation also charge transport and recombination play a crucial role in the devices, which were found out to become more and more an issue when going to finer mixed morphologies.

The findings of this study will provide a valuable tool for further research in the emerging field of hybrid solar cells, in particular, for optimizing polymer/nanocrystal hybrid solar cells using non-toxic material combinations including Sb_2_S_3_, Bi_2_S_3_ or CuInS_2_ nanocrystals. These materials also have the advantage of having better suited absorption properties for solar cell applications compared to CdS. Moreover, the nanomorphologies of hybrid absorber layers prepared with these materials in combination with a conjugated polymer still leave room for optimization, and thus, we are confident that the performed study will assist to improve the PCEs of in situ prepared hybrid solar cells.

## 4. Experimental Section

Cadmium(II) dithiocarbonate syntheses were carried out as follows: potassium hydroxide was stirred in a 1:1 molar ratio with a primary alcohol (e.g., 1-propanol to form propyl xanthate) and a small amount of water. The solution was cooled in an ice bath followed by a dropwise addition of carbon disulfide to a slight excess (1.1 equiv.). The resulting slurry was stirred for 30 min before vacuum filtering and washing with diethyl ether. All potassium xanthate products were then recrystallized from methanol. To form the cadmium salts, an aqueous solution of the respective potassium xanthate compounds was added to a rapidly stirring aqueous solution of cadmium nitrate in a molar ratio of 2:1. The products were filtered and washed with water followed by methanol. In the case of all except heptyl, pyridine adducts were obtained by adding 2 equiv. of pyridine to a suspension of the cadmium xanthate in dichloromethane, solubilising the compound, followed by evaporation of the solvent. All xanthates were then recrystallized from acetone. The heptyl xanthate was synthesized slightly differently and the according procedure can be found in the Supporting Information along with ^1^H NMR spectra for all products.

Typical thin films were produced by the spin coating of a chlorobenzene solution of P3HT (Sigma Aldrich, >98% RR, *M*_n_ 54–75 kDa) (9.375 mg ml^−1^) and Cd-ethyl-xanthate (150 mg ml^−1^). When using Cd-xanthate precursors with a longer ligand moiety a higher concentration was used to obtain the same final blend ratio.

Transmission electron microscopy (TEM) was performed using a JEOL 2000 MkII electron microscope operated at 200 kV. Films for TEM were fabricated on PEDOT:PSS (AI 4083, Ossila) itself spin coated onto glass to act as a water soluble sacrificial substrate. Free floating films were then transferred to copper grids for imaging in a ‘top-down’ fashion.

Steady-state absorption measurements were carried out with a Shimadzu 2600 spectrophotometer with an ISR-2600Plus Integrating Sphere Attachment. Photoluminescence measurements were made with a Horiba Jobin Yvon Fluorolog-2.

Femto-second TAS was measured on a previously described set-up[29] using pump and probe pulses generated from the output of a Solstice Ti:Sapphire regenerative amplifier (Spectra-Physics) with 800 nm 92 fs pulses with a 1 kHz frequency. The probe pulse was generated by a sapphire crystal and was split into a signal and a reference beam before the sample. The pump pulse of 570 nm was created using an optical parametric amplifier and a series of filters (TOPAS-NIRUVIS). It was then chopped mechanically to obtain a 500 Hz train of pulses. Both pump and probe pulses were focussed onto the same sample spot. TA spectra were calculated from the difference in detected probe light in presence and absence of the pump pulse.

Micro-second TAS measurements were performed by exciting the sample film under a dynamic nitrogen atmosphere using a dye laser pumped by a nitrogen laser. A 100 W quartz halogen lamp was used as a probe light source. The probe light passing through the sample film was detected with a silicon photodiode, amplified, and then measured using an oscilloscope. Full details of the setup can be found in the Supporting Information.

Thermogravimetric measurements were performed in helium atmosphere using a Netzsch Jupiter STA 449C (heating rate: 10 °C min^−1^). X-ray diffraction patterns were measured on a PANalytical X'Pert Pro MRD diffractometer using Ni filtered Cu K_α_ radiation at 40 kV and 40 mA. Simultaneous GISAXS and GIWAXS measurements were performed at the Austrian SAXS Beamline 5.2L of the electron storage ring ELETTRA (Italy)[35] using a similar setup as described before.[36] Details to the used setup can be found in the Supporting Information.

## Supporting Information

Supporting Information including details to experimental techniques, chemical syntheses and further data analysis (μs-TAS, GISAXS and GIWAXS setups, NMR data of synthetic products, and further analysis of GIWAXS and GISAXS data) is available from the Wiley Online Library or from the author.
