# Supplementary material for: Polymer/Nanocrystal Hybrid Solar Cells: Influence of Molecular Precursor Design on Film Nanomorphology, Charge Generation and Device Performance
Source: Adv Funct Mater. 2014 Nov 25;25(3):409–20. doi: 10.1002/adfm.201403108 (PMC4384757; doi:10.1002/adfm.201403108)
Supplement: Supplementary file 1 [file adfm0025-0409-sd1.pdf]

Copyright WILEY-VCH Verlag GmbH & Co. KGaA, 69469 Weinheim, Germany, 2014.

ADVANCED FUNCTIONAL MATERIALS

Supporting Information

for *Adv. Funct. Mater.*, DOI: 10.1002/adfm.201403108

Polymer/Nanocrystal Hybrid Solar Cells: Influence of Molecular Precursor Design on Film Nanomorphology, Charge Generation and Device Performance

Andrew J. MacLachlan, Thomas Rath, Ute B. Cappel, Simon A. Dowland, Heinz Amenitsch, Astrid-Caroline Knall, Christine Buchmaier, Gregor Trimmel, Jenny Nelson, and Saif A. Haque**

Copyright WILEY-VCH Verlag GmbH & Co. KGaA, 69469 Weinheim, Germany, 2013.

Supporting Information

Polymer/nanocrystal hybrid solar cells: Influence of molecular precursor design on film nanomorphology, charge generation and device performance

Andrew J. MacLachlan, Thomas Rath, Ute B. Cappel, Simon A. Dowland, Heinz Amenitsch, Astrid-Caroline Knall, Christine Buchmaier, Gregor Trimmel, Jenny Nelson, and Saif A. Haque**

Supporting Experimental

Micro-second transient absorption spectroscopy (μs-TAS) measurements were performed by exciting the sample film under a dynamic nitrogen atmosphere using a dye laser (Photon Technology International Inc. GL-301) pumped by a nitrogen laser (Photon Technology International Inc. GL-3300). A 100 W quartz halogen lamp (Bentham, IL 1) with a stabilized power supply (Bentham, 605) was used as a probe light source. The probe light passing through the sample film was detected with a silicon photodiode (Hamamatsu Photonics, S1722-01). The signal from the photodiode was amplified before being passed through electronic band-pass filters (Costronics Electronics). The amplified signal was collected with a digital oscilloscope (Tektronics, DPO3012), which was synchronized with a trigger signal from the pump laser pulse from a photodiode (Thorlabs Inc., DET210).

Simultaneous grazing incidence small and wide angle X-ray scattering (GISAXS, GIWAXS) measurements were performed at the Austrian SAXS Beamline 5.2L of the electron storage ring ELETTRA (Italy). For the GISAXS measurements, the beamline has been adjusted to a q-resolution ($q=4\pi/\lambda*\sin(2\theta/2)$, 2θ represents the scattering angle) between 0.1 and 3.1 nm^{-1} and the X-ray energy was 8 keV. The P3HT/cadmium xanthate samples were placed in a modified domed heating cell (DHS 1100 from Anton Paar GmbH, Graz, Austria) with a

grazing angle of about 0.18 ° and were heated from 35 °C up to 200 °C at a heating rate of approx. 10 °C min^{-1} under nitrogen atmosphere. During the temperature scan, data were recorded with 11 s time resolution using an image intensified CCD detector (GemStar/XIDIS model, Photonic Science Ltd., Millham, UK). For detection of the GIWAXS signal, a Pilatus 100K two-dimensional hybrid pixel array detector system (Dectris, Baden, Switzerland) was used. The angular calibration of the detectors was carried out using silver behenate powder with a d-spacing of 58.38 Å placed in a measuring capillary.

Further Information on Syntheses

Cadmium(II) O-ethyl dithiocarbonate

^{1}H NMR ($CDCl_3$, 400 MHz, δ): 1.50-1.47 (3H, t, CH_3), 4.54-4.49 (2H, q, CH_2), 7.48-7.45 (2H, m, *meta*CH), 7.88-7.83 (1H, m, *para*CH), 8.81-8.79 (2H, m, *ortho*CH) ppm.

Anal. calcd for $CdC_{16}N_2S_4O_2H_{20}$: C 37.50, H 3.91, N 5.47; found: C 37.52, H 3.92, N 5.59

Cadmium(II) O-propyl dithiocarbonate

^{1}H NMR ($CDCl_3$, 400 MHz, δ): 1.07-1.03 (3H, t, CH_3), 1.93-1.84 (2H, m, CH_2), 4.43-4.40 (2H, t, CH_2), 7.48-7.45 (2H, m, *meta*CH), 7.88-7.83 (1H, m, *para*CH), 8.81-8.79 (2H, m, *ortho*CH) ppm.

Anal. calcd for $CdC_{18}N_2S_4O_2H_{24}$: C 40.00, H 4.44, N 5.19; found: C 39.79, H 4.64, N 5.19

Cadmium(II) O-butyl dithiocarbonate

^{1}H NMR ($CDCl_3$, 400 MHz, δ): 1.01-0.98 (3H, t, CH_3), 1.55-1.45 (2H, m, CH_2), 1.88-1.81 (2H, m, CH_2), 4.48-4.45 (2H, t, CH_2), 7.50-7.47 (2H, m, *meta*CH), 7.90-7.85 (1H, m, *para*CH), 8.83-8.82 (2H, m, *ortho*CH) ppm.

Anal. calcd for $CdC_{20}N_2S_4O_2H_{28}$: C 42.25, H 4.93, N 4.93; found: C 41.99, H 5.04, N 4.77

Cadmium(II) O-pentyl dithiocarbonate

^{1}H NMR ($CDCl_3$, 400 MHz, δ): 0.97-0.94 (3H, t, CH_3), 1.43-1.36 (4H, m, CH_2CH_2), 1.90-1.83 (2H, m, CH_2), 4.47-4.44 (2H, t, CH_2), 7.50-7.47 (2H, m, *meta*CH), 7.90-7.85 (1H, m, *para*CH), 8.83-8.81 (2H, m, *ortho*CH) ppm.

Anal. calcd for $CdC_{22}N_2S_4O_2H_{32}$: C 44.30, H 5.37, N 4.70; found: C 44.02, H 5.58, N 4.64

Cadmium(II) O-2,2-dimethyl-3-pentyl dithiocarbonate

Cadmium (II) chloride (1.81 g, 9.86 mmol) was dissolved in 75 mL deionised water. Potassium O-2,2-dimethylpentan-3-yl dithiocarbonate (5.00 g, 21.7 mmol, 2.2 equiv.) which was synthesized according to literature[1] was dissolved in 75 mL of deionised water. The potassium xanthate solution was added dropwise to the cadmium chloride solution under stirring. The reaction was allowed to stir for about 2.5 hours and afterwards the white precipitate was filtered off and dried in vacuum. The precipitate was dissolved in chloroform and added to methanol to obtain a white powder, which was dried in vacuum (yield: 3.7 g, 77.8 %).

[1] Rath, T.; Edler, M.; Haas, W.; Fischereder, A.; Moscher, S.; Schenk, A.; Trattnig, R.; Sezen, M.; Mauthner, G.; Pein, A.; Meischler, D.; Bartl, K.; Saf, R.; Bansal, N.; Haque, S. A.; Hofer, F.; List, E. J. W.; Trimmel, G. *Adv. Energy Mater.* **2011**, *1*, 1046.

^{1}H NMR ($CDCl_3$, 300 MHz, δ): 5.00 – 4.95 (1H, m, CH), 1.80 – 1.66 (2H, m, CH_2), 0.99 – 0.95 (12H, m, 4xCH_3) ppm. ^{13}C NMR (75 MHz, $CDCl_3$, δ): 230.8 (C=S), 101.2 (CH), 36.1 (($C(CH_3)_3$)), 26.3 (3C, $C(CH_3)_3$), 23.6 (CH_2), 11.35 (CH_3-CH_2) ppm.

Anal. calcd for $C_{16}H_{3}0S_4O_2Cd$: C 38.82, H 6.11, S 25.90; found: C 38.84, H 6.11, S 25.91

^{1}H NMR and ^{13}C NMR spectra were recorded on a 300 MHz Bruker Ultrashield 300 or on a 400 MHz Bruker Avance spectrometer. Elemental analyses were carried out on a Universal CHNS Elemental Analyzer Vario El III.

Further Information on Solar Cell Fabrication

Devices fabricated all utilised an inverted architecture with the following layers: ITO/TiO_2(flat)/CdS(flat)/CdS:P3HT(blend)/MoO_3/Ag. ITO sputtered substrates (Psiotec Ltd) were sonicated in acetone, detergent, deionised water, and isopropanol. A solution of titanium isopropoxide (97%, Sigma Aldrich) and ethanolamine in 2-methoxyethanol was spun on substrates, which were then heated to 450 °C to form a flat TiO_2 layer. A solution of the Cd-ethylxanthate dissolved in chlorobenzene was deposited over the TiO_2 layer by spin coating and annealed under N_2 at 160 °C for 30 min in order to form the CdS layer. Chlorobenzene solutions of all the Cd-xanthates and P3HT (Sigma Aldrich) were mixed in a ratio so to keep a constant P3HT:CdS volume ratio of 1:1 across the range of precursors and maintain a consistent film thickness. These were then spin coated over the CdS layer followed by annealing at 160 °C for 1 h in an N_2 atmosphere. MoO_3 (10 nm) followed by Ag (100 nm) was evaporated on top.

Supporting Figures

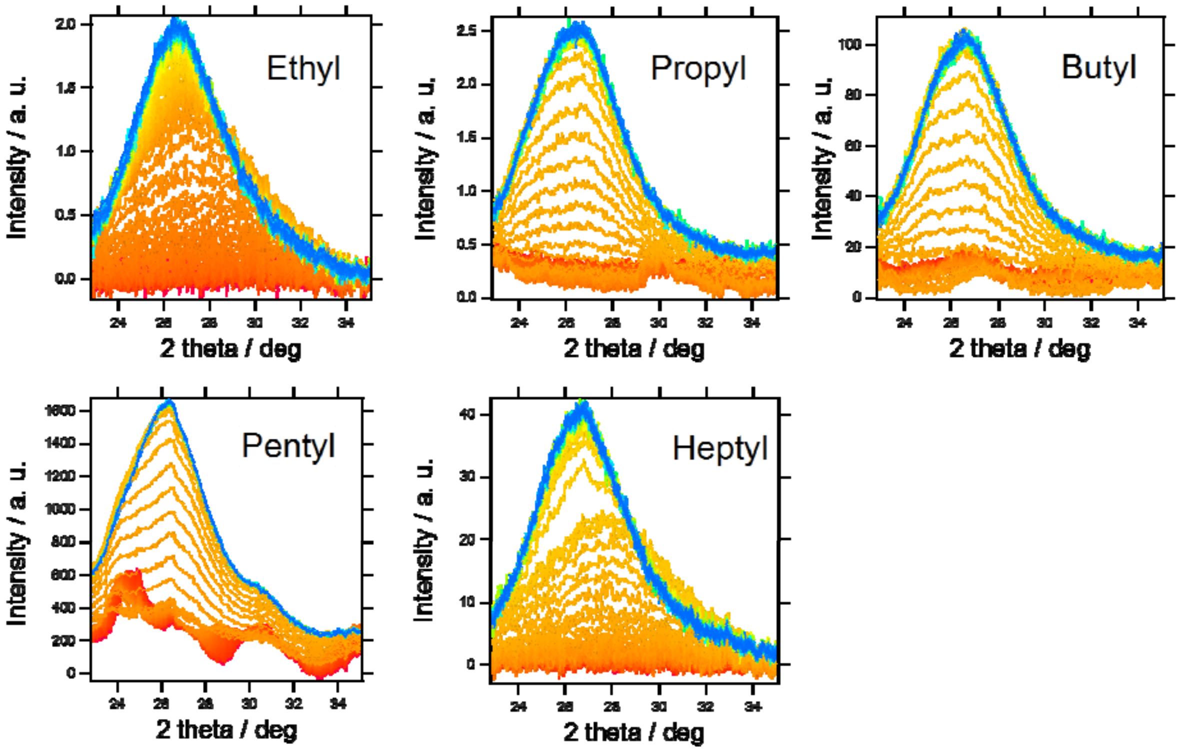

Figure S1. GIWAXS patterns of the investigated samples measured between 22.9 and 35 ° 2 theta during a heating run from room temperature to 200 °C (heating rate: 10 °C/min) showing the evolution of the most intense peak of CdS at around 27 ° 2 theta.

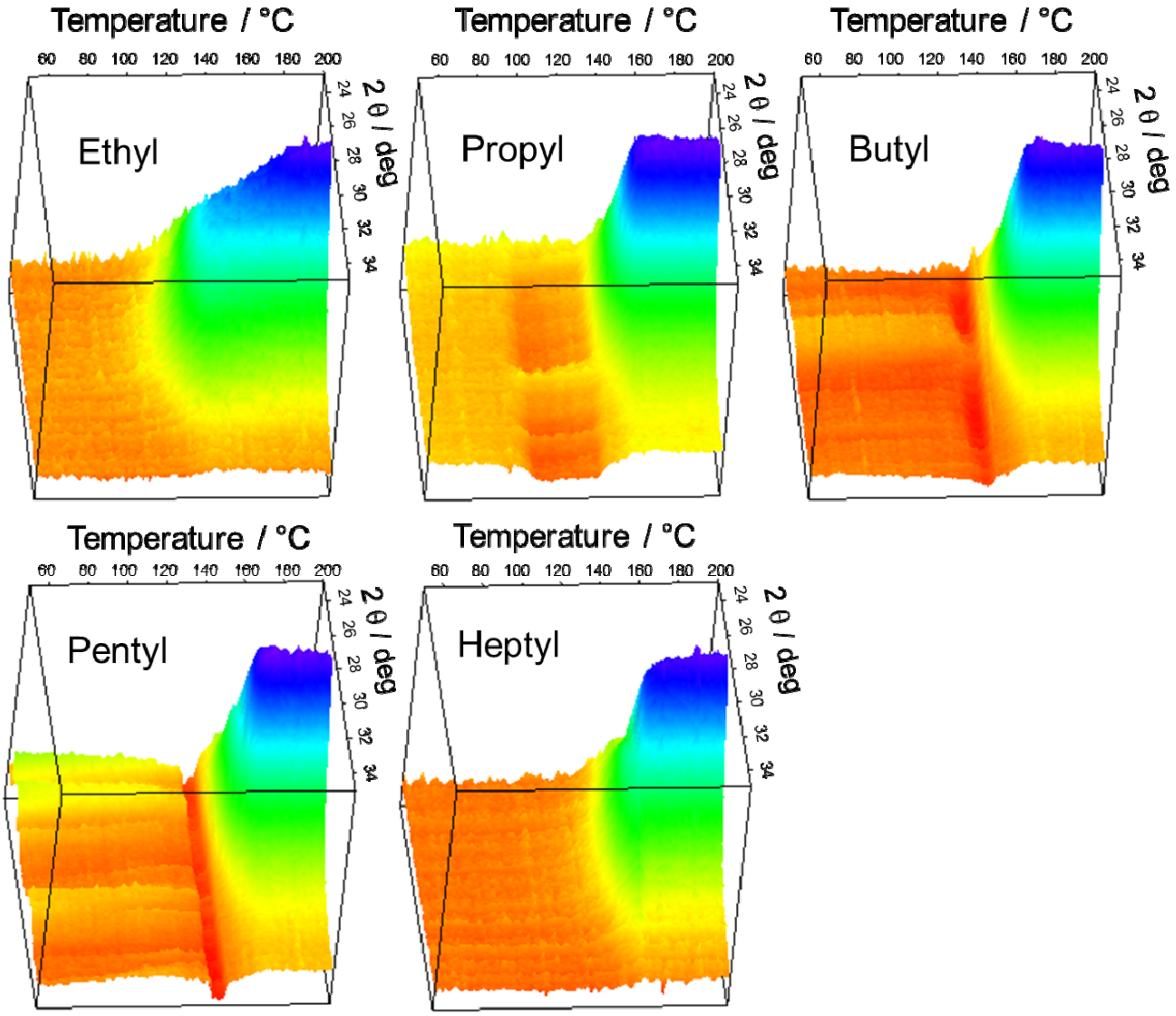

Figure S2. 3D plots showing the evolution of the most intense peak of the CdS phase with increasing temperature.

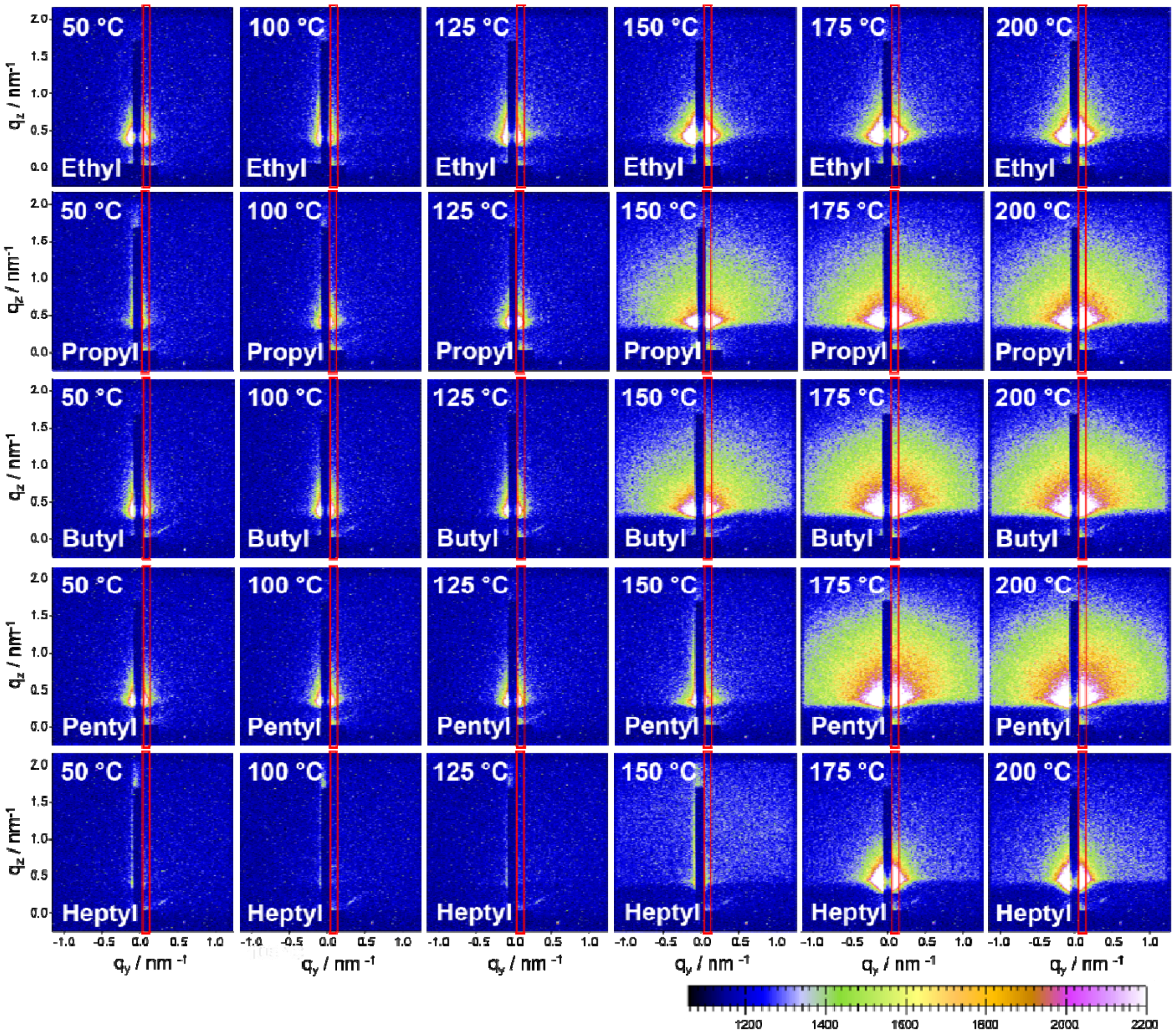

Figure S3. GISAXS images of the samples at different temperatures (50, 100, 125, 150, 175, 200 °C) during the heating run. The red boxes indicate the vertical areas used for integration.

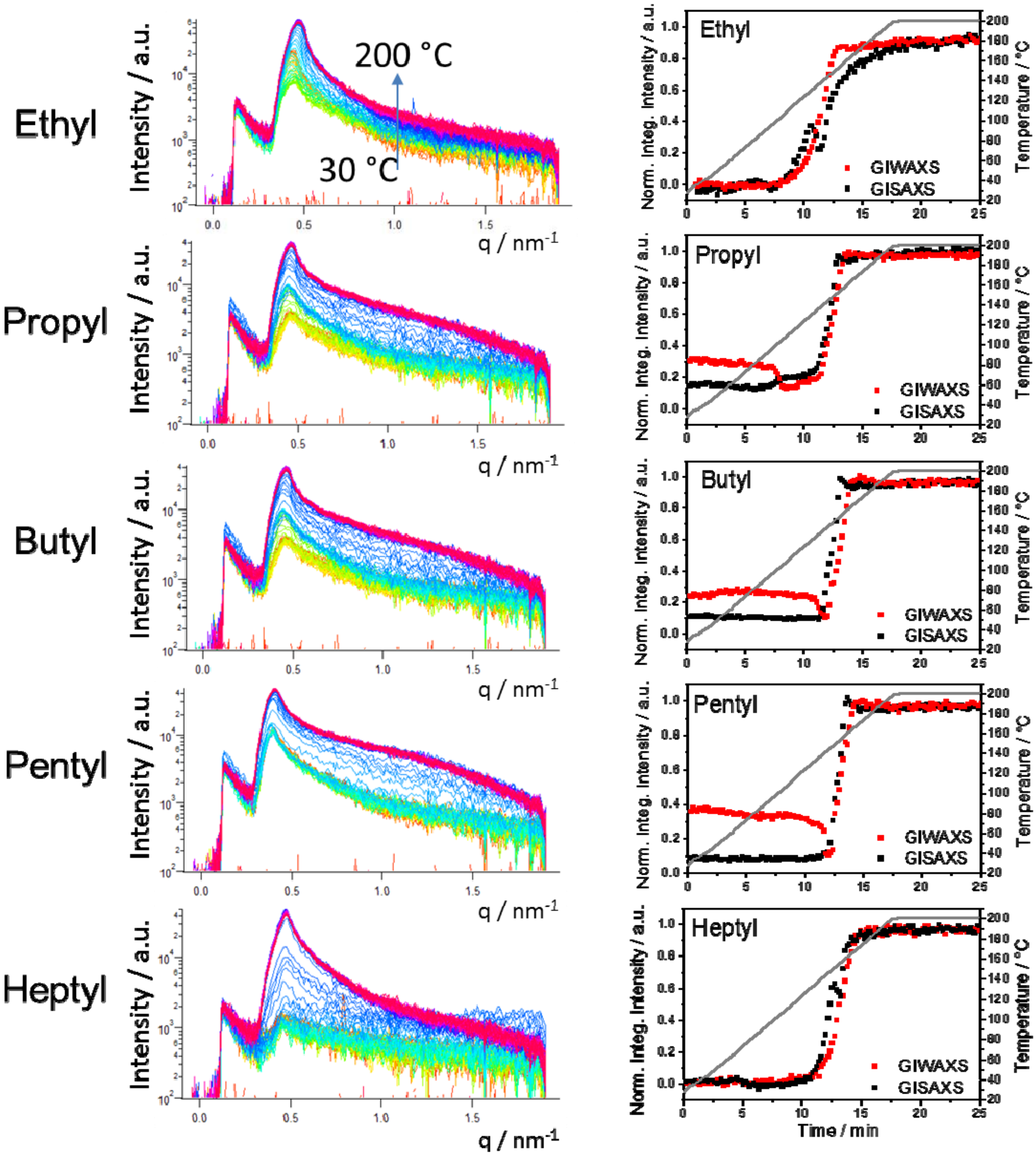

Figure S4. Left: Temperature-dependent evolution of the vertical cuts of the GISAXS patterns of the investigated samples. The increasing temperature (T) is indicated with an arrow in the Ethyl sample. Right: Integrated Intensities of the GISAXS patterns measured during the heating run compared with those of GIWAXS.

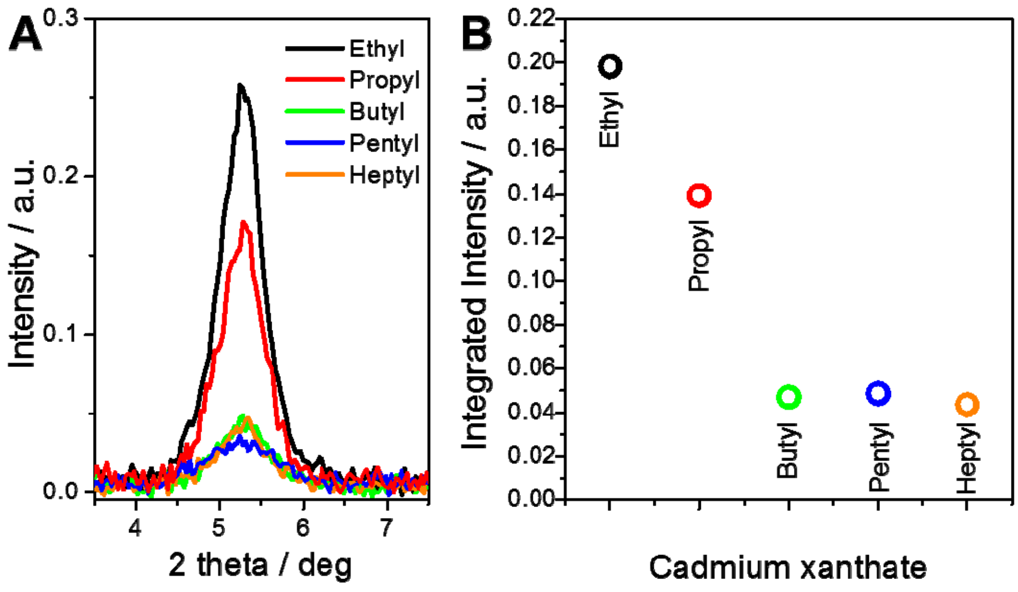

Figure S5. X-Ray diffraction patterns of the samples between 3.5 and 7.5 ° 2 theta showing different crystallinities of the P3HT phase in the hybrid layers (A) and the corresponding integrated intensities of the peak areas (B).
